# Medical Jurisprudence

**Published:** 1903-06

**Authors:** James R. Cain

**Affiliations:** Associate Member Chatham County Medical Society


### MEDICAL JURISPRUDENCE.

BY JAS. R. CAIN,
Associate Member Chatham County Medical Society.

GENTLEMEN:—By a coincidence this evening closes my eleventh year at the bar, as it also marks my first appearance before this body, composed of members of a sister profession; for Medicine, Law and Theology compose the trio of professions having most to do with humanity, shaping its course, meting out its happiness and stamping its destiny. For when sick or ailing we seek the doctor; when tangled or entoiled, the lawyer, and when troubled by conscience or bewildered in spirit, we seek a minister who may guide to hope, cheer and comfort.

Catching a suggestion from one of our members as to what line of discussion would likely interest this intelligent body of thinking men, I shall consider briefly three points.

1. What constitutes a contract or employment of a doctor.
2. The fees to which he is entitled.
3. Our collection laws in Georgia.

In other language we may state these heads thus:

1. How is a doctor employed?
2. How much can he charge?
3. How can he get his money?

In thus dealing with fundamentals I must forego discussing other branches of medical jurisprudence. In fact the subjects proposed are but phases of law in which doctors as business men are interested.

### CONTRACT OF EMPLOYMENT.

In our mother country, England, prior to the passage of the medical act (1858), a physician could not, as a general rule, recover for professional services in attending or prescribing for a patient. It seems that this rule was founded upon the general custom of the profession not to charge for professional services, the physician being presumed to practice in the expectation of an honorarium, not of remuneration; though if a physician acted in any other capacity than physician, he could maintain an action to recover for services provided he could show they were not rendered by him as a physician. A physician could, however, enter into a special agreement for the payment of his fees, and where such an agreement was made he was entitled to recover thereon. The prohibition (in England) against the recovery of compensation by a physician did not extend to surgeons or apothecaries. These were allowed to maintain an action to recover for medicine and attendance. (Ency. L. 22, 789.)

In the United States the rule has always been otherwise, and from a very early date it has been uniformly held that a physician may recover for services rendered by him. The employment or calling a physician without express agreement as to compensation raises an implied agreement on the part of the employer to pay for the services, on which implied agreement a *quantum meruit* will lie. (Ency. L. 22, 790.)

A physician may enter into a contract whereby his right to compensation will be made dependent upon his curing the patient entirely, or within a certain time. In such case failure to cure the disease forfeits his right to recover for services. The contract of employment of a doctor, like all other contracts, is the meeting of two minds on the same subject-matter at the same time. Express contracts, that is, those where all the terms are stated and are assented to, are plain and need no discussion. To entitle a physician to maintain an action against a person to recover for professional services rendered a third person, the physician must show a promise by the defendant, either express or implied, to pay therefor. (Ency. 22, 790.) The consent of the patient is not necessary to the validity of an express contract on the part of the third party to be answerable for a physician's compensation (Ency. 22, 790(10), nor is the liability of said third person affected by the fact that the liability is not assumed until after the physician had made several visits to the patient without the knowledge of such third person. (*Idem.*)

HOW MAY A CONTRACT OF EMPLOYMENT BE IMPLIED?

The necessity of the case or the urgent emergency may require a doctor, and if the circumstances demand a physician's being call ed, the person attended, his parent or guardian, shall pay for the services. No matter who may call a physician, the patient is liable therefor if he continues to receive the services. The same principle applies to parents, guardians and principals. Generally a doctor is called by an agent. Our Supreme Court recognizes this principle (47th Ga. 121), which was a case where the Central of Georgia Railway was sued for medical services rendered a negro boy who was run over by a train at Macon. When a physician is called, no matter by whom, the person is liable if he continue or receive the services after having a full knowledge of the facts.

Generally speaking, however, to bind one person for services rendered another, the relation of the patient to the person requesting services must be such as to raise the legal obligation on the part of the one charged to call a physician. The relation of parent and child, husband and wife, and in some cases of master and servant, are such relations as raise the presumption.

The Supreme Court of Indiana (9th L. R. A., p. 503) in 1890 made the following decision :

A physician and surgeon who, at the request of a general superintendent of a railway, renders professional services to the person injured by the company's trains in ignorance of the fact that the superintendent has no authority to contract for such services, may recover from the company, and the fact that the right to employ physicians and surgeons in such cases is vested in another officer, and that the company is not liable for the injuries which he was summoned to attend, are immaterial, since no inquiry was necessary in regard to such matters.

When the wife requests the services of a physician the presumption is that she acts as agent for her husband, the husband being the head of the household and liable for necessaries. If, however, she employs a physician on her own credit or separate estate, she would be held for charges. (Ency. 22, 792.)

The usual rules apply to contracts for medical services. To bind the principal, the services must be rendered upon his credit. A physician summoned in consultation by another physician may recover compensation from a patient for services, although there was an agreement between the attending physician and the patient that the former should be liable for the expenses of the consultation if the consulting physician did not assent to such understanding. (Ency. 22, 792, Subject, paragraph 5; 20th L. R. A. 698.)

GRATUITY.

While the law presumes a promise to pay for services of a physician, it is competent for the opposing party to show that the services were rendered gratuitously. The common understanding between the parties determine whether it was a gratuity. In absence of special contract or promise to pay, the law implies a promise to pay what the services are reasonably worth. (Ency. 22, 794.) In such case the physician must prove the ordinary and reasonable price for services of such a nature. He is not, however, obliged to prove the value of the services to the defendant. The customary charges of physicians of the neighborhood or locality may be shown by plaintiff or defendant. (Ency. 22, 794.) The skill and learning of the physician must be considered. (*Idem.*) The value is left to the jury. While in charge of a case a physician may make as many calls per day as his judgment and discretion may approve (Ency. 22, 794), though, of course, an express contract as to the number of calls may be made.

COLLECTION LAWS.

In Georgia an open account is valid and good for four years from maturity, that is, after it becomes due. Under the usages of this city when current bills are due the following month, an account is suable any time thereafter for four years from the first of the month following the date of services rendered. (Code 3424.)

In the distribution of assets, open accounts rank ninth on the list of priority, but the physician's bill for the last sickness is second and ranks with funeral expenses. For instance, if an estate was insolvent and the physician had attended the patient—the deceased—at sundry times during the four years preceding his death, he could only claim as a preferred account the bill for services for the last or fatal illness. Of course, if the estate was solvent, he would get his whole bill; otherwise he would only have the preferred claim for services rendered during the last illness. Open accounts bear interest at seven per cent. from maturity.

The books of a physician are admissible in evidence under certain conditions.. (Code 5182.) In case of accounts against deceased persons, the account should be itemized, that is, so stated as to show the exact days of each visit and the number of visits, and be sworn to and filed with the administrator or executor within twelve months from his qualification.

An interesting question recently arose in my practice as to the right to charge interest on an open account against an estate for the first year of administration. Under our statutes an administrator cannot be sued for the first year, during which time he has to ascertain the liabilities of the deceased and the assets to be administered. On careful investigation I concluded that there was no law in Georgia to compel an administrator to pay interest on an open account during the first year of administration. This much for the three subjects proposed in the beginning.

Another subject ever of interest to the medical profession is that of expert testimony and the rights of the physician to claim extra fees where he is called to testify as an expert.

We have no statute in Georgia on the subject. We have a provision for admitting expert testimony, but no provision for extra fees.

In three leading cases on the subject in other States, the trend of judicial opinion is against a physician in that they can be compelled to give expert testimony, even when no provision or offer is made to pay them special fees therefor.

In a leading case on the subject the Supreme Court of Arkansas (27th L. R. A. 669), has decided against the doctors in Flinn *vs.* Prairie County. It was as follows:

In 1894 Dr. B. W. Flinn, who was a physician, was summoned to testify on the part of the State in the criminal case pending in the Prairie circuit court, but on being called as a witness he asked the court to allow him fees as an expert before compelling him to testify. The court refused to make such allowance and required him to testify. Flinn afterwards presented to the county a claim for $150. In reviewing the decision of the lower court, the supreme court decided Dr. Flinn could not collect his fees. This case reviews a number of decisions of other States on the subject.

Perhaps the strongest case on this line is that of Dixon *vs.* People of Illinois. (168 Ill. 179, 39th L. R. A. 116.) This case was as follows:

January, 1895, in the circuit court of San Gammon county, Illinois, the case of Oliver Purdy against the city of Springfield was on trial. It was a suit for damages caused by a defective sidewalk. Dr. J. N. Dixon was called as an expert witness on the part of the city to testify. It was shown that he was a physician and surgeon, and that he had practiced as such twenty-one years, and nineteen of them in Springfield; that he was surgeon for five railroads running into the city, and had been such surgeon from two to seventeen years, and was a graduate of a regular school of medicine and had been practicing surgery for eighteen years. Dr. Dixon was requested to answer a hypothetical question propounded to him by the city's attorney. The witness declined to answer and stated the following reasons for refusing to answer, that is, on the grounds that an expert witness is entitled to a greater compensation than an ordinary witness is allowed; that an expert is not required to give expert testimony without compensation, as an expert without reasonable compensation has been paid. My fee as an expert in this case is ten dollars. Witness said, I have not been paid or offered any compensation for my professional testimony in this case, nor has such compensation been provided for. But on the contrary, it has been expressly refused me; therefore I decline to testify until the fee is provided for. It was admitted that the witness knew nothing of the facts in the case. It was also admitted that the charge of ten dollars as a fee was a fair and reasonable one, but the city had no authority to pay such fee and had not promised to pay the same. The witness was brought into court by a regular subpœna as any other witness. The witness was ordered by the court to answer the question and he again declined, when the clerk was directed to docket a case against the doctor for contempt. He was then fined twenty-five dollars for contempt of court for refusing to answer. From this judgment the appeal was taken to the supreme court. The supreme court of Illinois seems to have reviewed the law of all the States on the subject and decided against the doctor and sustained the fine. In the course of its reasoning the court uses this language: "The question in this case is whether a physician who is subpœnaed as a witness only can be punished as for a contempt for refusing to testify when no compensation greater than that allowed an ordinary witness has been paid or promised to him. Where a physician is required to make an application of his knowledge to a particular case, say as to secure a particular result, such as, for instance, the curing of a disease or the healing of a wound, then he would undoubtedly be entitled to a compensation. A physician or surgeon cannot be punished for a contempt for refusing to make a post-mortem examination unless paid therefor; nor can he be required to prepare in advance for testifying in court by making an examination or performing an operation, or resorting to a certain amount of study without being paid therefor, but when he is required to answer an hypothetical question which involves a special knowledge of his calling he is merely required to do what every good citizen is required to do in behalf of public peace, public order, and in promotion of the public good. It is the purpose of the ordinary witness and the expert witness to testify as to facts within his knowledge which bear upon the decision in the courts. Such duty devolves upon him as a citizen and in view of the protection which he receives from the law of the land in the protection of his person and property. He is required to testify as an expert witness in answer to hypothetical questions as does the ordinary witness to testify to facts within his knowledge." (This decision cites, ex parte Dement, 53 Ala. 389.) That case holds that the law allowed no excuse for withholding evidence which is relevant to the matters in question before its tribunals. The same principle which justifies calling the mechanic from his workshop, merchant from his storehouse, the broker from his change, or the lawyer from his engagements to testify to some matter which he has learned in the exercise of his art or profession, authorizes the summoning of a physician or surgeon or skilled apothecary to testify of a like matter. When relevant to the case pending for determination in the judicial tribunals, the physician, like any other person, may be called to testify as an expert in a judicial investigation, whether it be of a civil or criminal nature, without being paid for his testimony as for professional opinion. Upon refusal to testify he is punishable as for a contempt. This decision of the Alabama court has been followed by the States of Minnesota, Texas, Colorado and Arkansas. Thus by analogy, in absence of a statute on the subject, it is possible that a physician in Georgia might be compelled to give expert testimony without being previously tendered expert fees for such.

